# Assessment of macular microvascular changes in children following treatment of anisometropic myopic amblyopia using optical coherence tomography angiography

**DOI:** 10.1007/s00417-023-06055-8

**Published:** 2023-04-13

**Authors:** Heba Radi AttaAllah, Sahar Torky Abdelrazik Abdelaziz, Asmaa Anwar Mohamed Mohamed, Mohamed Farag Khalil Ibrahiem

**Affiliations:** 1 Ophthalmology department, Faculty of Medicine, Minia University, Minia, Egypt; 2 Ophthalmology department, Minia University Hospital, Minia University, Minia, 61111 Egypt

**Keywords:** OCTA, Anisometropic amblyopia, Vessel density, FAZ, Amblyopia treatment, Microvascular changes

## Abstract

**Purpose:**

To evaluate macular microvascular changes in the form of foveal avascular zone (FAZ) area and vessel density in the superficial, deep capillary plexuses, and choriocapillaris using optical coherence tomography angiography (OCTA) in children with anisometropic myopic amblyopia before and after treatment.

**Methods:**

This prospective observational study included 32 patients younger than 12 years old with anisomyopic amblyopia. OCTA was done before patients’ treatment with optical correction with or without patching and was repeated after successful amblyopia treatment. Outcomes included superficial, deep, and choriocapillaris vessel density (VD) and superficial and deep FAZ areas.

**Results:**

The study included 13 males (40.6%) and 19 females (59.4%), and the mean age was 9.52 ± 1.33 years. Fifty-three percent (53%) of patients needed only optical correction, and the remaining 47% needed additional patching therapy. After successful treatment, there was a significant improvement in amblyopic eyes in best-corrected visual acuity (*p* < 0.001), with higher VD values in superficial capillary plexuses (*p* < 0.001), deep capillary plexuses (*p* < 0.001), and foveal choriocapillaris (*p* = 0.030). In the glasses with patching subgroup, the difference between pre-treatment and post-treatment parameters revealed a significant improvement in vessel density in superficial retinal plexuses (foveal and parafoveal; *p* values 0.023 and < 0.001, respectively) and deep retinal plexuses (whole image, foveal, and parafoveal; *p* values 0.003, < 0.001, and 0.002, respectively). While amblyopic eyes treated with glasses alone had a significantly greater difference in choriocapillaris foveal VD (*p* value = 0.022).

**Conclusion:**

After effective amblyopia treatment, amblyopic eyes exhibited improved best-corrected visual acuity and better macular perfusion along the superficial, deep vascular density, and choriocapillaris foveal VD.

**Clinical trial registration:**

CinicalTrials.gov Identifier: NCT05223153.


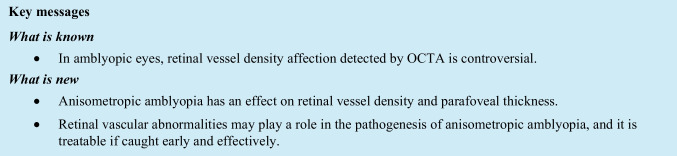


## Introduction

Abnormal visual stimulation during early visual development results in amblyopia. It is characterized by reduced best-corrected visual acuity (BCVA) in the absence of clinically detectable fundus abnormalities. It affects about 2–4% of children. Uncorrected refractive error, strabismus, and deprivation amblyopia are the main causes of amblyopia [1, 2].

The pathogenesis of amblyopia is still unknown, though the lateral geniculate body and visual cortex have been shown to be affected in several types of amblyopia [3].

Recent research indicates that retinal vascular anomalies may also be involved. Noninvasive ophthalmologic imaging using optical coherence tomography (OCT) and optical coherence tomography angiography (OCTA) were used to detect macular changes, visualize, and quantify the retinal blood vessels in anisometropic, strabismic, mixed, and/or ametropic amblyopia with no conclusive results [4, 5].

Although many studies using OCTA to evaluate amblyopic eyes (strabismic, anisometropic, and/or ametropic) found lower retinal vessel density in the superficial and deep capillary plexuses compared to normal eyes [6–9], others found no changes in retinal vessel density [10].

Few studies have investigated the effect of amblyopia treatment on macular perfusion and compared pre- and post-treatment vessel density values in strabismic, anisometropia, or combined amblyopia [11, 12].

To the best of our knowledge, this is the first study to evaluate macular microvascular changes in vessel density and the foveal avascular zone (FAZ) area in the superficial, deep capillary plexuses, and choriocapillaris using OCTA in children with anisometropic myopic amblyopia only before and after treatment with glasses and/or patching.

Because of the variability in treatment outcomes of anisometropic amblyopia [13], anisometropic myopic amblyopia was chosen for this study. Effective retinal blood perfusion is crucial for maintaining normal visual function, and in myopic eyes, axial stretching and elongation may have an impact on this perfusion [14], with lower vessel density in the deep capillary plexus being detected in myopia [15]. Furthermore, this decrease in vessel density may impair best-corrected visual acuity and reduce contrast sensitivity [16].

## Methods

This prospective observational study included 32 patients with anisometropic amblyopia attending the outpatient clinic of the Ophthalmology Department, Minia University Hospital, between September 2021 and September 2022. The study followed the principles of the Helsinki Declaration and was approved by the Minia University Faculty of Medicine’s Institutional Review Board (Approval number: 7692021). All children’s parents provided informed written consent for their children’s participation in the study.

Young patients less than 12 years old with unilateral anisomyopic amblyopia, with myopia less than − 6.00 diopters, and without previous treatment for the amblyopia were included in the study.

Patients with bilateral amblyopia, strabismic or deprivational amblyopia, retinal disease, uncooperative patients, patients with media opacity impairing retinal visualization, poor fixation resulting in motion artifacts, images with a low signal strength index (SSI less than 50), or significant blink artifacts were excluded from the study.

A full ophthalmological examination was done, including best-corrected visual acuity (BCVA) assessment using the Snellen chart (measurements were converted to logarithms of the minimum angle of resolution (log MAR) equivalents for data analysis), anterior segment examination using slit lamp biomicroscopy, cycloplegic refraction using cyclopentolate eye drops, orthoptic, and fundus examination. Amblyopia is defined as a difference in best-corrected visual acuity between the two eyes of two lines on an acuity chart [17].

All patients had moderate to severe amblyopia at the time of the first visit assessment [17].

Full cycloplegic refraction was prescribed for all patients.

The Compact Touch A-B scan (Quantel Medical, Cournon d'Auvergne, France) was used to determine the axial length of the globe.

All patients started with their prescribed optical correction as an initial treatment for anisometropic amblyopia [18] and were followed up every 4 weeks to detect BCVA improvement.

At the follow-up visits, if the interocular difference in BCVA ≤ 1 line, patients were considered to have resolved amblyopia and continued to wear their spectacle correction, and did not receive patching treatment.

If the interocular difference in BCVA was greater than two lines after 16 weeks, patients required additional occlusion therapy (occlusion of the sound eye with an opaque adhesive patch for 6 h per day) in addition to their optical correction. Occlusion was maintained in order to achieve equal visual acuity in both eyes [13].

Amblyopic eyes were divided into two subgroups based on the type of treatment (glasses and glasses with patching).

Optical coherence tomography angiography (OCTA) was performed on each patient at the time of amblyopia diagnosis (prior to treatment) and after treatment (at the patient’s final visit during the study when amblyopia was resolved).

### Optical coherence tomography angiography OCTA evaluation

The Avanti RTVue-XR system with optical software (Angio Vue version 2017.1.0.155; Optovue, Inc.) was utilized for OCTA imaging, which uses a split-spectrum amplitude-decorrelation angiography (SSADA) approach to retrieve OCT angiography information.

The Angio Retina protocol was used to acquire OCTA images, using a scan area of 6 × 6 mm^2^.

Automated segmentation was used for the definition of different vascular plexuses: the superficial capillary plexus (SCP) is identified between 3 μm below the internal limiting membrane (ILM) and 15 μm below the inner plexiform layer (IPL), the deep capillary plexus (DCP) is located between 15 and 70 μm below the IPL, and the choriocapillaris is found between 30 and 60 μm below the RPE reference.

AngioAnalytics are measurement tools that allow for the assessment of vascular area density (VD) and non-flow area in order to determine the size of the FAZ. The vessel density at the level of the superficial and deep retinal capillaries, as well as the choriocapillaris, was assessed and analyzed using a vessel density map, and defined as the relative density of flow as a proportion of the total area. After image acquisition, the FAZ area was measured in square millimeters (mm^2^) at the level of the superficial and deep retinal plexuses using the non-flow feature of the OCTA software. The area of the FAZ is automatically estimated by the program (RTVue-XR version: 2017.1.0.151) when the operator taps on the center of the FAZ.

In amblyopic eyes, measured VD values and FAZ areas were corrected by the magnification factor of the image according to the following formula:

$${D}_{t}^{2}/{D}_{m}^{2}={0.002066\left(\mathrm{AL }- 1.82\right)}^{2}$$

(where *D*_*t*_ is the true size of the measured retinal feature, *D*_*m*_ is the measured size on the OCTA image, and AL is axial length) [19].

The best-corrected visual acuity, foveal and parafoveal thickness, vessel density (VD), and FAZ area were compared using OCTA at three levels (superficial, deep, and choriocapillaris) between the sound and amblyopic eyes.

Also, a comparison was made between the amblyopic eyes before and after treatment with either glasses or glasses with patching.

### Statistical method

The data was coded, tabulated, and statistically analyzed using the Statistical Package for Social Sciences (SPSS) program (software version 25; SPSS Inc., IBM Corp., New York, USA, 2017).

Descriptive statistics were done for parametric (normally distributed) quantitative data by mean, standard deviation (SD), and minimum and maximum of range and for qualitative data by frequency and percentage.

Analyses were done between the two groups for parametric quantitative data using the independent samples *t* test, and for non-parametric quantitative data using the Mann–Whitney *U* test, while analyses between the two times within each group for parametric quantitative data were done using the paired samples *t* test.

Analyses were done for qualitative data using the chi square test.

Pearson’s correlation was done between continuous quantitative variables.

The level of significance was taken at *P* value ≤ 0.05.

## Results

Thirty-eight patients with unilateral anisometropic amblyopia met the inclusion criteria. Thirty-two patients were compliant with their optical correction and were included in the study.

The age range was 7–12 years (mean ± SD = 9.52 ± 1.33). There were 13 (40.6%) males and 19 (59.4%) females.

The range of refraction in the sound eyes was − 0.25/0.25 diopters (mean ± SD = 0 ± 2) while it was − 4/ − 6 diopters (mean ± SD =  − 4.9 ± 0.7) in the amblyopic eyes (*p* < 0.001) (Table 1).

**Table 1:** Pre-treatment visual acuity, refraction, foveal thickness, parafoveal thickness, FAZ area, and VDs at different levels in sound and amblyopic eyes

1^st^ visit		Sound	Amblyopic	*P* value
		*N* = 32	*N* = 32	
Age of patients (years)	Range Mean ± SD	7–12 9.52 ± 1.33		
Log MAR BCVA	Range Mean ± SD	(0–0) 0 ± 0	(0.7–1) 0.8 ± 0.1	< 0.001*
Refraction (*D*)	Range Mean ± SD Median/IQR	(− 0.25:0.25) 0 ± 2 0/(− 0.25:0.25)	(− 6: -4) − 4.9 ± 0.7 − 4.9/(− 5.5: − 4.3)	< 0.001*
Foveal thickness	Range Mean ± SD	(224–255) 245.7 ± 10.2	(236–258) 244.4 ± 6.9	0.547
Parafoveal thickness	Range Mean ± SD	(294–312) 301 ± 5.2	(297–339) 313.6 ± 17.1	< 0.001*
Superficial FAZ (mm^2^)	Range Mean ± SD	(0.13–0.4) 0.23 ± 0.1	(0.15–0.45) 0.26 ± 0.12	0.206
Deep FAZ (mm^2^)	Range Mean ± SD	(0.2–0.41) 0.28 ± 0.07	(0.23–0.45) 0.31 ± 0.09	0.161
Superficial whole VD (%)	Range Mean ± SD	(47.2–54.6) 51.8 ± 2.4	(47.8–51.8) 49.2 ± 1.6	< 0.001*
Superficial foveal VD (%)	Range Mean ± SD	(27.2–42.3) 37.6 ± 5.5	(26.2–44.3) 36.2 ± 6.5	0.368
Superficial parafoveal VD (%)	Range Mean ± SD	(47.6–54.9) 52.1 ± 2.4	(47.7–53.6) 50.6 ± 2	0.008*
Deep whole-image VD (%)	Range Mean ± SD	(52.4–62.1) 58.6 ± 3.4	(50.7–63.2) 57 ± 4.4	0.097
Deep foveal VD (%)	Range Mean ± SD	(30.6–45.1) 40 ± 5.9	(25.8–46.9) 39.2 ± 7.2	0.635
Deep parafoveal VD (%)	Range Mean ± SD	(57.7–66.6) 64.2 ± 2.9	(57.6–68.1) 63.3 ± 3.4	0.258
Choriocapillaris whole-image VD (%)	Range Mean ± SD	(63.6–69.5) 67.4 ± 1.8	(63.9–70.7) 65.7 ± 2.1	< 0.001*
Choriocapillaris foveal VD (%)	Range Mean ± SD	(61.6–69.6) 65.4 ± 2	(61–68.5) 63.7 ± 2.3	0.003*
Choriocapillaris parafoveal VD (%)	Range Mean ± SD	(61.4–72) 65.8 ± 2.4	(62.6–71.9) 65.3 ± 2.8	0.432

Independent samples* T* test for parametric quantitative data

Mann–Whitney *U* test non-parametric quantitative data

^*^Significant level at *P* value < 0.05

*Log MAR BCVA* logarithm of best-corrected visual acuity, *D* diopters, *VD* vessel density, *FAZ* foveal avascular zone, *SD* standard deviation

Seventeen patients out of 32 (53.13%) had a good response to their correction, with improvement in the mean best-corrected visual acuity to 0 ± 0.1 log MAR after 12–16 weeks (mean ± SD = 14.12 ± 1.62) and the remaining 15 (46.87%) required additional occlusion therapy in addition to their correction, and the mean best-corrected visual acuity was 0.1 ± 0.1 log MAR after a mean follow-up period of 25.4 ± 3.31 (range, 20–30 weeks).

Table 1 shows significantly higher parafoveal thickness in amblyopic eyes before treatment than in sound eyes (*p* value < 0.001). Also, superficial whole-image vessel density (VD), superficial parafoveal VD, and choriocapillaris (whole image and foveal) VD had significantly lower values than sound eyes (*p* values < 0.001, 0.008, < 0.001, and 0.003 respectively).

**Fig. 1:**
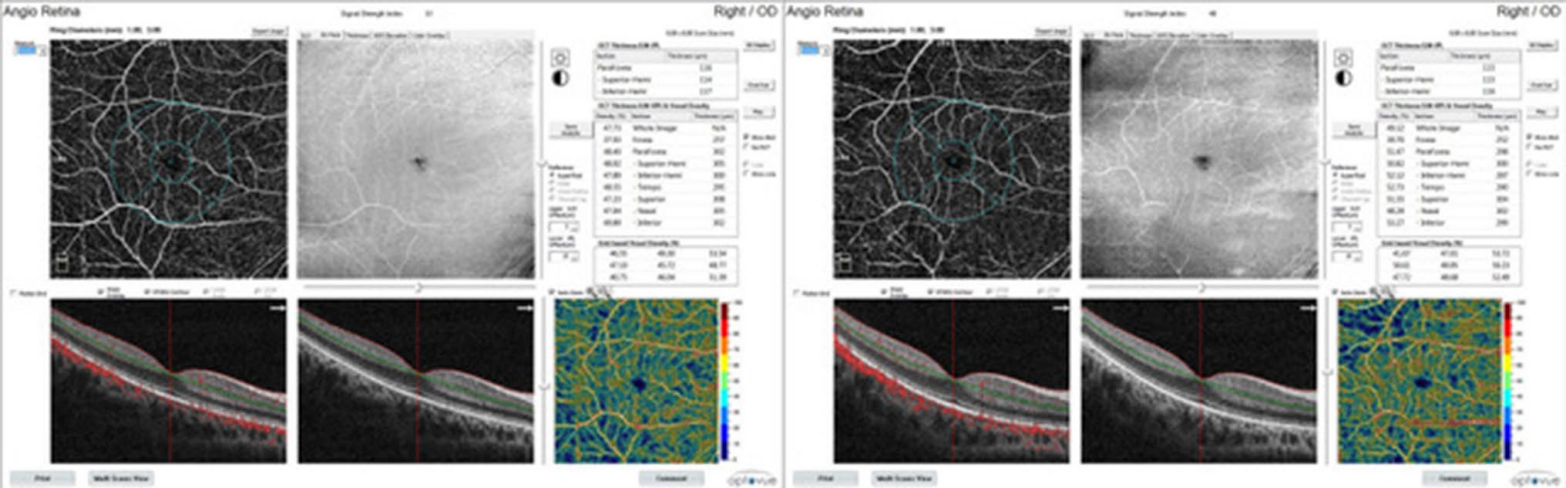
Retina scan (6 × 6 mm^2^) at the level of the superficial retinal plexus centered on the macula in an OCTA angio of the right eye of a patient with amblyopia (middle of the right). The OCT thickness ILM-RPE and vessel density map, grid-based vessel density (percent) with color-coded flow density map (bottom right), shows the vessel density (left image: pre-treatment, right image: improvement of the vessel density after amblyopia treatment)

There was a significant improvement in amblyopic eyes in best-corrected visual acuity (*p* < 0.001), with higher VD values in superficial capillary plexuses (whole, foveal, and parafoveal, *p* < 0.001), deep capillary plexuses (whole image, foveal, and parafoveal, *p* < 0.001), and foveal choriocapillaris (*p* = 0.030) after treatment (Fig. 1). While superficial FAZ showed significant improvement (*p* = 0.020) (Table 2).

**Table 2:** Best-corrected visual acuity, refraction, FAZ area, and VD at different levels in amblyopic eyes before and after treatment

Amblyopic eye		Pre-treatment	Post-treatment	*P* value
		*N* = 32	*N* = 32	
Refraction (*D*)	Range Mean ± SD	(− 6.00: − 4.00) − 4.9 ± 0.7	(− 6.00: − 4.00) − 4.9 ± 0.7	1
Log MAR BCVA	Range Mean ± SD	(0.7–1) 0.8 ± 0.1	(0–0.2) 0.1 ± 0.1	< 0.001*
Superficial FAZ (mm^2^)	Range Mean ± SD	(0.15–0.45) 0.26 ± 0.12	(0.13–0.36) 0.22 ± 0.09	0.020*
Deep FAZ (mm^2^)	Range Mean ± SD	(0.23–0.45) 0.31 ± 0.09	(0.24–0.44) 0.31 ± 0.07	0.894
Superficial whole VD (%)	Range Mean ± SD	(47.8–51.8) 49.2 ± 1.6	(49.9–52.8) 51 ± 1.1	< 0.001*
Superficial foveal VD (%)	Range Mean ± SD	(26.2–44.3) 36.2 ± 6.5	(31.2–45.2) 39.5 ± 5.4	< 0.001*
Superficial parafoveal VD (%)	Range Mean ± SD	(47.7–53.6) 50.6 ± 2	(50.3–55.6) 53.2 ± 1.5	< 0.001*
Deep whole-image VD (%)	Range Mean ± SD	(50.7–63.2) 57 ± 4.4	(53.1–64.1) 60.5 ± 2.6	< 0.001*
Deep foveal VD (%)	Range Mean ± SD	(25.8–46.9) 39.2 ± 7.2	(35.3–47.9) 43.3 ± 4.6	< 0.001*
Deep parafoveal VD (%)	Range Mean ± SD	(57.6–68.1) 63.3 ± 3.4	(63.1–78) 66.4 ± 2.9	< 0.001*
Choriocapillaris whole-image VD (%)	Range Mean ± SD	(63.9–70.7) 65.7 ± 2.1	(64.2–68.9) 65.9 ± 1.4	0.717
Choriocapillaris foveal VD (%)	Range Mean ± SD	(61–68.5) 63.7 ± 2.3	(60.3–69.1) 64.7 ± 3.1	0.030*
Choriocapillaris parafoveal VD (%)	Range Mean ± SD	(62.6–71.9) 65.3 ± 2.8	(64.3–68.8) 66.1 ± 1.4	0.209

Paired samples *T* test

^*^Significant level at *P* value < 0.05

*D* diopters, *Log MAR BCVA* logarithm of best-corrected visual acuity, *VD* vessel density, *FAZ* foveal avascular zone, *SD* standard deviation

In Table 3, amblyopic subgroups (glasses and glasses with patching) were compared before treatment as regards best-corrected visual acuity, refraction, and OCTA parameters.

**Table 3:** Comparison of best-corrected visual acuity, refraction, FAZ area, and VDs between 2 subgroups of amblyopic eyes before treatment

Amblyopic eye Pre-treatment		Glasses	Glasses + patching	*P* value
		*N* = 17	*N* = 15	
Refraction (*D*)	Range Mean ± SD	(− 6.00: − 4.00) − 4.7 ± 0.5	(− 6.00: − 4.00) − 5.2 ± 0.7	0.011*
Log MAR BCVA	Range Mean ± SD	(0.7–1) 0.8 ± 0.1	(0.7–1) 0.9 ± 0.1	0.010*
Superficial FAZ (mm^2^)	Range Mean ± SD	(0.15–0.44) 0.22 ± 0.09	(0.15–0.45) 0.32 ± 0.13	0.015*
Deep FAZ (mm^2^)	Range Mean ± SD	(0.23–0.43) 0.27 ± 0.06	(0.23–0.45) 0.35 ± 0.1	0.010*
Superficial whole VD (%)	Range Mean ± SD	(47.8–51.8) 49.6 ± 1.9	(47.8–50.6) 48.8 ± 1.2	0.179
Superficial foveal VD (%)	Range Mean ± SD	(35.2–44.3) 40 ± 4.1	(26.2–39.7) 31.9 ± 6.2	< 0.001*
Superficial parafoveal VD (%)	Range Mean ± SD	(48.4–53.6) 51.5 ± 2	(47.7–51.4) 49.5 ± 1.5	0.004*
Deep whole-image VD (%)	Range Mean ± SD	(51.7–63.2) 59.4 ± 3.8	(50.7–58.6) 54.2 ± 3.2	< 0.001*
Deep foveal VD (%)	Range Mean ± SD	(40.3–46.9) 43.8 ± 2.9	(25.8–44) 34 ± 7.1	< 0.001*
Deep parafoveal VD (%)	Range Mean ± SD	(61.2–68.1) 65.6 ± 2.3	(57.6–64.4) 60.7 ± 2.3	< 0.001*
Choriocapillaris whole-image VD (%)	Range Mean ± SD	(64.9–65.5) 65 ± 0.2	(63.9–70.7) 66.5 ± 2.9	0.049*
Choriocapillaris foveal VD (%)	Range Mean ± SD	(61–64.1) 62.6 ± 1.4	(62.3–68.5) 65 ± 2.4	0.001*
Choriocapillaris parafoveal VD (%)	Range Mean ± SD	(62.7–65.1) 64.5 ± 0.7	(62.6–71.9) 66.3 ± 3.8	0.060

Independent samples *T* test

^*^Significant level at *P* value < 0.05

*D* diopters, *Log MAR BCVA* logarithm of best-corrected visual acuity, *VD* vessel density, *FAZ* foveal avascular zone, *SD* standard deviation

Before treatment, amblyopic eyes treated with glasses had significantly higher best-corrected visual acuity and lower refractive error, with significantly higher VDs values in superficial (foveal and parafoveal) and deep (whole image, foveal, and parafoveal) retinal plexuses with smaller superficial and deep FAZ areas when compared with amblyopic eyes treated with glasses and patching. On the other hand, the glasses and patching subgroup had higher significant whole image and foveal choriocapillaris vessel density.

After treatment, amblyopic eyes treated with glasses and patching had significantly lower VDs values in superficial (foveal), deep (foveal and parafoveal) retinal plexuses with larger superficial and deep FAZ areas when compared with amblyopic eyes treated with glasses only (Table 4).

**Table 4:** Comparison of best-corrected visual acuity, refraction, FAZ area, and VDs between 2 subgroups of amblyopic eyes after treatment

Amblyopic eye Post-treatment		Glasses	Glasses + patching	*P* value
		*N* = 17	*N* = 15	
Refraction (*D*)	Range Mean ± SD	(− 6.00: − 4.00) − 4.7 ± 0.5	(− 6.00: − 4.00) − 5.2 ± 0.7	0.011*>
Log MAR BCVA	Range Mean ± SD	(0–0.2) 0 ± 0.1	(0–0.2) 0.1 ± 0.1	0.002*
Superficial FAZ (mm^2^)	Range Mean ± SD	(0.13–0.33) 0.17 ± 05	(0.15–0.36) 0.27 ± 0.1	0.001*
Deep FAZ (mm^2^)	Range Mean ± SD	(0.25–0.43) 0.27 ± 0.04	(0.24–0.44) 0.35 ± 0.08	0.003*
Superficial whole VD (%)	Range Mean ± SD	(49.9–52.1) 50.8 ± 1	(49.9–52.8) 51.3 ± 1.1	0.234
Superficial foveal VD (%)	Range Mean ± SD	(31.2–45.2) 42.3 ± 3.4	(31.2–45.2) 36.3 ± 5.5	0.001*
Superficial parafoveal VD (%)	Range Mean ± SD	(50.3–54.1) 52.9 ± 1.2	(50.3–55.6) 53.6 ± 1.8	0.192
Deep whole-image VD (%)	Range Mean ± SD	(53.1–63.6) 61.2 ± 2.6	(57.4–64.1) 59.7 ± 2.3	0.086
Deep foveal VD (%)	Range Mean ± SD	(37.4–47) 45.6 ± 2.3	(35.3–47.9) 40.6 ± 5.2	0.001*
Deep parafoveal VD (%)	Range Mean ± SD	(63.7–78) 67.6 ± 3	(63.1–69.1) 65.1 ± 2.1	0.008*
Choriocapillaris whole-image VD (%)	Range Mean ± SD	(64.2–68.9) 66.2 ± 1.8	(64.8–66) 65.5 ± 0.5	0.118
Choriocapillaris foveal VD (%)	Range Mean ± SD	(60.3–69.1) 64.2 ± 3.7	(61–67.5) 65.2 ± 2.2	0.404
Choriocapillaris parafoveal VD (%)	Range Mean ± SD	(64.4–68.8) 66.5 ± 1.6	(64.3–67.3) 65.7 ± 1	0.082

Independent samples *T* test

^*^Significant level at *P* value < 0.05

*D* diopters, *Log MAR BCVA* logarithm of best-corrected visual acuity, *VD* vessel density, *FAZ* foveal avascular zone, *SD* standard deviation

When pre-treatment and post-treatment parameter differences in two subgroups were compared, amblyopic eyes treated with glasses and patching demonstrated statistically significant differences (higher improvement) in vessel density in superficial retinal plexuses (foveal and parafoveal; *p* values 0.023, < 0.001 respectively) and deep retinal plexuses (whole image, foveal, and parafoveal; *p* values 0.003, < 0.001 and 0.002 respectively). While amblyopic eyes treated with glasses alone had a statistically significant difference in choriocapillaris foveal VD (*p* value = 0.022) (Table 5).

**Table 5:** Pre- and post-treatment difference in best-corrected visual acuity and OCTA parameters in amblyopic subgroups treated with glasses and glasses with patching

Difference (pre-post)		Glasses	Glasses + patching	*P* value
		*N* = 17	*N* = 15	
Refraction (*D*)	Median IQR	0 (0:0)	0 (0:0)	1
Log MAR BCVA	Median IQR	0.7 (0.7:0.8)	0.7 (0.7:0.8)	0.444
Superficial FAZ (mm^2^)	Median IQR	0 (0:0.1)	0.1 (0:0.1)	0.126
Deep FAZ (mm^2^)	Median IQR	0 (0:0)	0 (0:0.1)	0.112
Superficial whole VD (%)	Median IQR	− 2 (− 2.2: − 0.3)	− 2 (− 2.6: − 1.9)	0.135
Superficial foveal VD (%)	Median IQR	− 0.8 (− 6.1: − 0.8)	− 4.5 (− 6.5: − 3.3)	0.023*
Superficial parafoveal VD (%)	Median IQR	− 1.9 (− 2.3: − 0.4)	− 4.3 (− 5.8: − 2.1)	< 0.001*
Deep whole-image VD (%)	Median IQR	− 1.3 (− 2.8: − 0.3)	− 6.4 (− 6.5: − 2.1)	0.003*
Deep foveal VD (%)	Median IQR	− 1.6 (− 4:0)	− 6.6 (− 9.6: − 5.6)	< 0.001*
Deep parafoveal VD (%)	Median IQR	− 1.1 (− 1.9: − 0.2)	− 5.5 (− 5.6: − 2.4)	0.002*
Choriocapillaris whole-image VD (%)	Median IQR	− 0.3 (− 4:0)	− 0.5 (− 1.2:4.9)	0.450
Choriocapillaris foveal VD (%)	Median IQR	− 0.4 (− 4.9:0.1)	0.3 (− 0.1:1)	0.022*
Choriocapillaris parafoveal VD (%)	Median IQR	− 1.4 (− 3.7: − 0.7)	− 1.5 (− 1.9:6.6)	0.326

Mann–Whitney *U* test

^*^Significant level at *P* value < 0.05

*D* diopters, *Log MAR BCVA* logarithm of best-corrected visual acuity, *VD* vessel density

In amblyopic eyes, there was a positive moderate correlation between refraction and post-treatment OCTA parameters in the following areas: superficial foveal VD (*r* = 0.701, *p* < 0.001); deep whole-image VD (*r* = 0.588, *p* < 0.001); deep foveal VD (*r* = 0.711, *p* < 0.001); and fair correlation with deep parafoveal VD (*r* = 0.463, *p* = 0.008).

On the other hand, there was a moderate negative correlation between refraction and post-treatment superficial FAZ area (*r* =  − 0.694, *p* < 0.001) and deep FAZ area (*r* =  − 0.689, *p* value < 0.001).

Furthermore, there was a negative correlation between pre-treatment log MAR BCVA and post-treatment vascular parameters (higher VD after treatment with lower log MAR values, or better visual acuity) in the following areas: superficial foveal VD (*r* =  − 0.695, *p* value < 0.001), deep whole-image VD (*r* =  − 0.605, *p* value < 0.001), deep foveal VD (*r* =  − 0.717, *p* value < 0.001), and deep parafoveal VD (*r* =  − 0.370, *p* value = 0.037).

## Discussion

The effect of amblyopia treatment using OCTA has been discussed in a few previous studies that included amblyopic eyes of different etiologies (strabismic, anisometropic, or mixed), and changes in vessel density within the superficial and deep retinal capillary plexuses were reported [11, 12, 20].

To the best of our knowledge, this is the first longitudinal study to evaluate changes in FAZ area (superficial and deep) and vessel density not only at the level of the retinal capillary plexuses (superficial and deep), but also at the choriocapillaris, following amblyopia treatment in children with anisometropic amblyopia.

### Foveal and parafoveal thickness

Amblyopic eyes showed a significant increase in parafoveal thickness (*p* < 0.001) compared with their sound eyes, without a significant difference in foveal thickness between the two groups. The latter finding was consistent with the findings of Singh et al. and Taskiran Comez et al., who reported a nonsignificant difference in central macular thickness (CMT) between better and worse eyes with anisomyopia [21, 22]. In contrast to our findings, Rajavi and his co-workers reported that amblyopic eyes had increased macular retinal thickness at the foveal area (1-mm ring) than fellow eyes and controls, and no significant difference was found at the 3 mm ring (parafoveal) [23], this could be attributed to various inclusion criteria for their research participants, as they included patients with varying degrees of amblyopia (mild, moderate, and severe), whereas the current study included children with moderate and severe amblyopia.

### Vessel density (VD) in the superficial, deep, and choriocapillaris plexus before treatment

Before treatment, there was a statistically significant decrease in vascular density in amblyopic eyes at the level of the superficial retinal plexuses (whole image and parafoveal) compared with fellow eyes. This finding was consistent with the findings of Hamurcu and colleagues, who found a statistically significant decrease in whole and parafoveal superficial capillary plexus (SCP) vessel density in amblyopic eyes compared to healthy controls [24].

Although the choroid primarily nourishes the external retina, neurovascular coupling has demonstrated how a light stimulus can modify the vascular parameters of the superficial plexuses that nourish the internal retina [25], suggesting that the lower VD in the superficial capillary plexus in amblyopic eyes could be due to improper development due to a lack of normal visual experience [8].

Previous studies found a significantly lower vessel density in the deep retinal plexus as well as the superficial retinal plexus in amblyopic eyes, and this difference could be explained by comparing the amblyopic eyes to healthy controls rather than their fellow eyes [8]. Furthermore, amblyopic eyes in their studies had various etiologies (ametropic, strabismic, anisometropic, and meridional amblyopia) [7], and strabismic amblyopia in Yilmaz et al. [9].

In the current study, there was a significant decrease in vessel density at the choriocapillaris level (whole image and foveal) in the amblyopic anisomyopic eyes compared with the sound fellow eye. This finding is consistent with previous observations obtained by Liu et al., who concluded that when anisometropia exceeded 1.50 *D*, the choriocapillaris vascular density in the more myopic eyes was considerably lower than in the less myopic eyes [26].

In contrast to our findings, Borrelli and colleagues discovered significantly increased choriocapillaris vessel density in amblyopic eyes (anisometropic and strabismic) when compared to control eyes, but not in the same patient’s sound eye. Furthermore, their patients were hypermetropic (mean refractive error was 4.3 ± 6.2), with a thicker retina and increased vessel density as a result [27].

### Effect of amblyopia treatment on OCTA parameters

There was a wide variation in OCTA findings in amblyopia with diverse aetiologies. Therefore, we chose one form of amblyopia (anisomyopic amblyopia) to be specific in our results regarding this type and avoid any variation with different types of amblyopia.

To the best of our knowledge, this is the first study to compare the effects of effective amblyopia therapy on OCTA measures such as FAZ area alterations and vessel density changes at the level of the retinal plexus and choriocapillaris in anisomyopic patients to the same amblyopic eyes before treatment.

After proper and successful treatment of amblyopia with glasses alone or glasses and patching, a significant improvement was reported in amblyopic eyes in terms of best-corrected visual acuity (*p* < 0.001), with higher VD values in superficial and deep capillary plexuses (whole, foveal, and parafoveal, *p* < 0.001 for all), and choriocapillaris (foveal, *p* = 0.030).

Similar findings were reported by Gunzenhauser and colleagues, who found a significant increase in VD values in some regions of their study (whole image and superior hemisphere) at the level of deep capillary plexus and superficial foveal vessel density after treatment using 3 × 3-mm OCTA scans, despite the fact that their study was conducted on patients with strabismic amblyopia, and they suggested that the increase in retinal vessel density could be attributed to strabismus surgery in strabismic patients [11]. Our patients were anisometropic, treated with glasses only or with patching, so the possibility of improved retinal vessel density due to surgery was excluded from our explanations.

Also, Salerni et al. found a higher macular vascular density at the level of superficial plexuses in successfully treated amblyopic patients than in unresponsive amblyopic and normal eyes, as their study only investigated vessel density at the superficial retinal plexuses, and participants had different types of amblyopia (strabismus, anisometropia, and meridional) [20].

Zhang and his associates examined the effect of treatment on the retinal vessel density in anisometropic amblyopia by comparing newly diagnosed amblyopes with treated amblyopes and controls and found a significant reduction in superficial and deep vessel density compared with controls and a significantly lower superficial vessel density between amblyopes and treated amblyopes, but they did not compare pre- and post-treatment vessel density [12].

As the optical treatment of amblyopia should be the first line of treatment in patients with refractive error [28], glasses were prescribed for all our patients. Fifty-three (53.12%) of the patients had best-corrected visual acuity 0–0.2 log MAR with glasses after 12–16 weeks. Forty-seven (46.88%) patients needed occlusion therapy with their glasses, and improvement in the mean best-corrected visual acuity reached 0.1 ± 0.1 log MAR after a mean follow-up period of 25.4 ± 3.31 weeks. This finding is in line with that of Cotter and colleagues, who discovered that by 9 weeks, about half of the children had reached their best acuity [29]. However, the length of time depends on the patients' compliance with glasses wear [30].

To our knowledge, this is the first research to identify the effect of optical correction alone on OCTA parameters, while the few earlier studies only identified the effect of patching treatment with glasses if required [11, 12, 20].

Amblyopic eyes were divided into two subgroups according to treatment (glasses and glasses with patching) and compared before treatment as regards best-corrected visual acuity, refractions, and OCTA parameters. Amblyopic eyes treated with glasses had a significantly lower refraction and better best-corrected visual acuity with smaller superficial and deep FAZ areas when compared with amblyopic eyes treated with glasses and patching. Furthermore, the VD values in the superficial (foveal and parafoveal) and deep (whole image, foveal, and parafoveal) retinal plexuses were significantly higher, which could be the major reason for these eyes’ rapid recovery with only optical treatment, as Huang and his co-workers suggested that the clearer visual stimulation caused by optical therapy may promote the process of macular vascular pruning in the amblyopic eyes, thereby improving macular perfusion and increasing FAZ circularity [31]. Wang and Xia also discovered that a decrease in vessel density could contribute to poor best-corrected visual acuity [16].

On the other hand, there was higher vessel density in the whole image and foveal choriocapillaris in patients treated with glasses with patching (Table 3).

When the difference between pre-treatment and post-treatment parameters in two subgroups was compared, amblyopic eyes treated with glasses and patching showed greater improvement in vessel density in superficial capillary plexuses (foveal and parafoveal) and deep capillary plexuses (whole image, foveal, and parafoveal), and this finding may be attributed to a longer time of treatment in the patching subgroup with additional stimulation to photoreceptors and, as a result, increased metabolic and oxygen requirements for this layer, which is supplied in part by the deep capillary plexus (10–15%) [32].

Amblyopic eyes treated with glasses alone improved more in choriocapillaris foveal VD (Table 5).

In amblyopic eyes, there was a positive correlation between refraction and post-treatment superficial foveal VD, deep whole-image VD, and deep foveal and parafoveal VD.

In the current study, all amblyopic eyes were myopic. Myopia may lead to the narrowing and straightening of blood vessels [33]. Treatment for amblyopia in myopic eyes may stimulate the retinal layers’ need for more oxygen, resulting in increased blood circulation.

On the other hand, there was a moderate negative correlation between refraction and post-treatment superficial and deep FAZ areas, which means that amblyopic eyes with higher degrees of myopia in our study had larger FAZ areas. This might be explained by the fact that a thicker retina has a greater metabolic need, which is correlated with a decrease in the FAZ area and vice versa [34]. Myopic eyes already have a thinner retina and, as a result, a greater FAZ.

Before treatment, amblyopic eyes’ log MAR best-corrected visual acuity was negatively correlated with post-treatment improvement in superficial foveal VD, deep capillary plexus VD (whole image, foveal, and parafoveal), (higher VD after treatment with lower log MAR values, or better visual acuity). As a result, severe amblyopia results in less recovery of vessel density following successful anisomyopic amblyopia treatment.

Although the purpose of this study was to evaluate microvascular changes after amblyopia treatment in eyes with anisomyopic amblyopia to avoid variable factors and detect specific results, it has several limitations, including a small number of patients, and participants who are younger and have a better response to amblyopia treatment cannot be included as they are uncooperative. Further research with a larger number of patients and diverse age groups is required in each type of amblyopia separately, as well as in anisometropic amblyopia due to different refractive errors.

In conclusion, amblyopic eyes had higher parafoveal thickness and lower superficial and choriocapillaris VD than sound eyes before treatment, which was significantly improved after visual acuity improvement in both glasses and glasses with patching subgroups. Following successful anisomyopic amblyopia treatment, severe amblyopia results in less recovery of the retinal vessel density.
